# Vestibular rehabilitation in a university hospital

**DOI:** 10.1016/S1808-8694(15)31095-8

**Published:** 2015-10-19

**Authors:** Flávia da Silva Tavares, Maria Francisca Colella dos Santos, Keila Alessandra Baraldi Knobel

**Affiliations:** 1 Speech therapist specialized in human communication disorders, São Paulo Federal University (Universidade Federal de São Paulo), UNIFESP/EPM. Specializing in gerontology, São Paulo Federal University, UNIFESP/EPM; 2 Doctor. Professor in the Speech Therapy course, Campinas State University (Universidade Estadual de Campinas), UNICAMP; 3 Doctoral student in otorhinolaryngology, USP Medical School. Responsible for vestibular rehabilitation in the Otoneurology Unit, Clinical Hospital, Campinas State University (Universidade Estadual de Campinas), HC/UNICAMP

**Keywords:** vestibular rehabilitation, dizziness, vertigo

## Abstract

The aim of vestibular rehabilitation is to improve total balance, quality of life and spatial orientation of patients with dizziness.

**Aims:**

To determine the characteristics of the patients who underwent the Vestibular Rehabilitation program of the Neurotology Ward of a University Hospital, and to verify the results obtained between November/2000 and December/2004.

**Materials and Methods:**

analysis of 93 files from patients under Vestibular Rehabilitation during the studied period.

**Study design:**

Retrospective clinical.

**Results:**

the mean age of patients was 52.82 years, 56 females and 37 males. The average number of therapy sessions was 4.3, higher for patients with central neurotological disorders (average of 5.9). Among the patients who concluded the treatment, 37 (60.7%) had significant improvement, 14 (22.9%) presented partial improvement and 10 (16.4%) did not report significant benefits. Patients with peripheral neurotological disorders were the ones who most benefited from Vestibular Rehabilitation.

**Conclusion:**

Most of the patients were female, with a mean age of 52.8 years. Fifty one patients (83.6%) benefited from the therapy, confirming treatment efficacy.

## INTRODUCTION

Three systems that collect information about the external environment, namely vision, proprioception and the vestibular system, assure normal balance. The vestibular system provides information about angular head acceleration in different spatial planes (sagittal, axial and coronal planes) and linear bodily movements (forward, backward, upward and downward). Vision is responsible for the rapid assimilation of body movements and for the sense of depth. The proprioceptive system includes structures located in muscles, tendons, joint capsules and cutaneous tissues that provide information about the position of various bodily segments in space in any given instant. All of this information is sent to the central nervous system (CNS) to be analyzed, compared and integrated.^1^ Sensory conflict occurs when information provided to the nervous centers is not coherent, giving rise to vertigo and unbalance.^2^

Dizziness is the illusion of movement by an individual or his or her surrounding environment. Dysfunction in any of the bodily balance segments may cause this symptom. The most common organic cause of vertigo is damage of the vestibular system.^3^

There are many causes of dizziness, the most common being benign paroxysmal postural vertigo (BPPV), vestibular neuritis, Ménière's disease, perilymphatic fistulae, circulatory, metabolic, hormonal and immunological conditions, alterations of the cervical spine, cranial trauma and psychoaffective disorders.^4^, ^5^, ^6^

Some of the causes are common in certain age groups. Children commonly present infectious labyrinthic diseases (otitis, viral disease), benign paroxysmal vertigo, trauma, ototoxicity and kinetosis. After age 20 years, common conditions are neuronitis, hormonal and metabolic labyrinthic diseases and Ménière's disease. Hormonal and metabolic dysfunctions are more common in women. After age 50 years, the etiology is commonly linked with vascular conditions and some cervical syndromes.^4^

Aging leads to structural degeneration of the three systems involved in maintaining body balance (visual, proprioceptive and vestibular systems) and their corresponding reflexes. Examples are: there are fewer sensory labyrinthic cells and vestibular nerve fibers, vision is affected by glaucoma or cataract, muscle mass is lost, ligaments and tendons become less flexible, degenerative arteritis and osteoporosis arise, and bodily movements become more difficult, leading to physical inactivity.

Unbalance is one of the main limiting factors in elderly people; no specific cause is found in 80% of cases. Dizziness affects daily activities in about 20% of people aged over 60 years; patients may fall and suffer fractures. Fear of falling is one of the causes of falls in elderly persons. Fear leads to a limitation of daily activities, with resulting losses in family, social and professional relationships. Vestibular Rehabilitation (VR) is one of the most effective methods for recovering bodily balance in elderly patients.^7^

Labyrinthic dysfunction may be treated by at least three approaches: medication, surgery and VR.

VR is therapy that aims for vestibular compensation through specific and repeated exercises that activate neural plasticity mechanisms in the CNS.^8^, ^9^, ^10^

When there is vestibular injury, CNS neuroplasticity leads to functional recovery of body balance. This adaptive mechanism of vestibular motor behavior is named vestibular compensation. There may also be adaptation, habituation and/or substitution. VR accelerates these mechanisms, thus reducing vestibular symptoms.^11^^,^^12^

In adaptation, the vestibular system learns to receive and process distorted or incomplete information, which is then made appropriate to the stimuli. Vestibular habituation consists of a reduction in sensory responses based on the repetition of sensory stimuli. It is attained by executing repeated movements that reduce the vestibular response, which in turn decreases the amplitude of nystagmus (rhythmic and involuntary oscillation of the eyes). Maximum integration of visual, vestibular and proprioceptive sensors is required for habituation to occur. Repetition facilitates adaptation to movement and stimulates sensorial organs, thus generating new automatic responses in the database that is responsible for bodily balance.^11^^,^^13^ Vestibular substitution occurs when absent or conflicting information about bodily balance is substituted.^11^

Another mechanism is restitution, which is total repair following limited and temporary injury. It may occur if there is acute labyrinthic inflammation or infection, for instance. Once the causative agent ceases operating, patients are cured and free from the complaints.^9^

An intervention based on specific, repeated and prolonged exercises is required if spontaneous recovery of adaptive vestibular motor behavior mechanisms is incomplete, to foster CNS neuroplasticity; this is the task of VR.^9^

We believe that a study of the results attained by VR in a teaching hospital is paramount, considering the limitations of body balance disorders and the feasibility, low cost and extremely low rate of side effects of VR.

The purposes of this study were to define the profile of patients seen at the Vestibular Rehabilitation Outpatient Unit of the Otoneurology Sector in a university hospital, to assess the outpatient unit itself and to evaluate its results, between November 2000 and December 2004.

## MATERIAL AND METHOD

This study was a retrospective investigation, approved by the Research Ethics Committee of a university (protocol number 369/02). Data were collected from the files of patients that had done VR in the Vestibular Rehabilitation Outpatient Unit of the Otoneurology Sector in a university hospital between November 2000 and December 2004.

All patients underwent an otorhinolaryngological evaluation, audiological testing and electronystagmography. VR sessions were individual and included the following: hearing and balance function related to the labyrinthic alteration of the patient, guidance about VR itself (labyrinthic compensation mechanisms, neuroplasticity and function of exercises) and exercises based on Cawthorne and Cooksey's protocol.^14^ Exercises were used according to the needs of each patient. If necessary, exercises for further adaptation using balls, gyration, cards and mattresses were applied. Physicians applied the repositioning maneuvers for BPPV patients, which therefore were not included as part of VR.

All data, except for exam results, were obtained from information provided by patients themselves in the clinical history. Information not in these charts was sought for in the archived files in the hospital. Some of the items in our protocol were not found in the outpatient charts or in the hospital files, and were not filled in.

The following data were collected: age, sex, result of the audiological evaluation (classified according to Lloyd and Kaplan^15^), result of electronystagmography (classified by otorhinolaryngologists who were responsible for the test as normal, peripheral, central, mixed or inconclusive), approximate time elapsed between the initial complaint of dizziness and referral do VR, psychoaffective complaints (anxiety, depression and insecurity), general health (arterial hypertension, spinal disorders, metabolic conditions, strokes, cranial trauma, problems with vision, and others), result of VR (classified as significant improvement, partial improvement, unsatisfactory improvement and quitting), and number of VR sessions.

Discharge criteria for therapy sessions were based on reports by patients of improvement from dizziness, of stabilization in the progression of disease, or of noncompliance to the proposed treatment.

Results of VR were classified according to the reports given by patients on the degree of discomfort due to dizziness at the beginning and end of therapy (on a 1 to 10 visual-analog scale) and speech therapist observations. Patients who visited our unit only once or who did not return on expected dates were classified as “quitters.,”

Data were codified, digitized and analyzed in the SPSS (Statistical Package Social Science) software, version 11.0. Student's t test, Spearman's correlation coefficient, the Kruskal-Wallis test and the chi-square test were used for the statistical analysis. A results was statistically significant when “p,” was less than 5% (p<0.05).

## RESULTS

The sample was composed of 93 files of patients seen at the VR outpatient unit. Table 1 shows the distribution of subjects by sex and the mean age (with the standard deviation of age).

**Table 1:** Distribution of subjects by sex, mean age, and the standard deviation of age (N=93).

Sex	N	%	Mean age	Standard deviation
Female	56	60.2%	54.0	14.9
Male	37	39.8%	50.9	15.2
Total	93	100%	52.8	15.0

**Figure 1:**
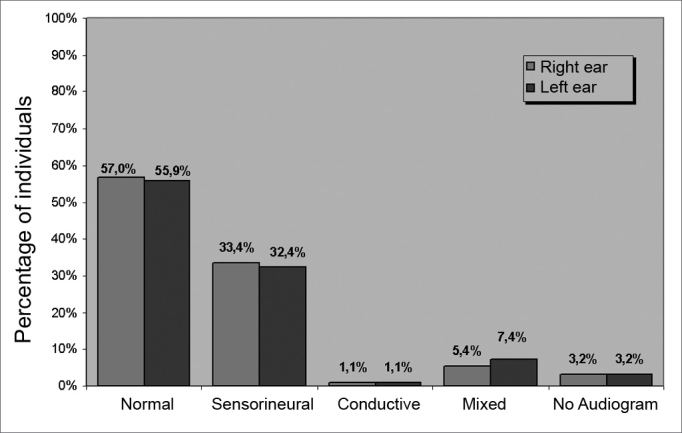
Distribution of subjects according to the type of hearing loss for each ear.

Figure 1 shows the results of the audiological assessment classification according to the type of hearing loss.

Table 2 shows the results of electronystagmography according to sex.

**Table 2:** Distribution of subjects according to the electronystagmography and sex (N= 93).

	Female		Male		Total	
	N	%	N	%	N	%
Normal	13	14.0%	4	4.3%	17	18.3%
Peripheral	31	33.3%	25	26.9%	56	60.2%
Central	7	7.5%	4	4.3%	11	11.8%
Mixed	1	1.1%	1	1.1%	2	2.2%
Inconclusive	4	4.3%	3	3.2%	7	7.5%
Total	56	60.2%	37	39.8%	93	100.0%

Table 3 shows the time elapsed between the onset of dizziness and the beginning of VR.

**Table 3:** Distribution of subjects in time elapsed between the onset of dizziness and the beginning of VR (N=93).

Time	N	%
1 to 5 years	64	68.8%
6 to 10 years	13	14.0%
Over 10 years	16	17.2%
Total	93	100.0%

**Figure 2:**
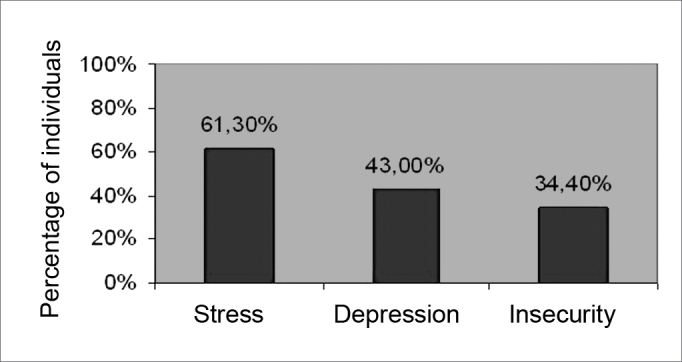
Distribution of subjects according to the occurrence of psychoaffective complaints.

Figure 2 shows the psychoaffective complaints.

Table 4 shows the general health status and the percentage of other diseases in the sample group.

**Table 4:** Distribution of subjects according to the main health complaints.

Complaint	N	%
Metabolic	20	21.5%
Vision	17	18.3%
Spine	50	53.8%
Head trauma	17	18.3%
AH	31	33.3%
CVA	6	6.5%
Other	61	65.6%

**Key:**

Metabolic: Metabolic conditions

Vision: Problems with vision

Spine: Vertebral spine problems

Head trauma: Cranial trauma

AH: Arterial Hypertension

CVA: Stroke or Cerebral Vascular Accident

Others: Other health

**Figure 3:**
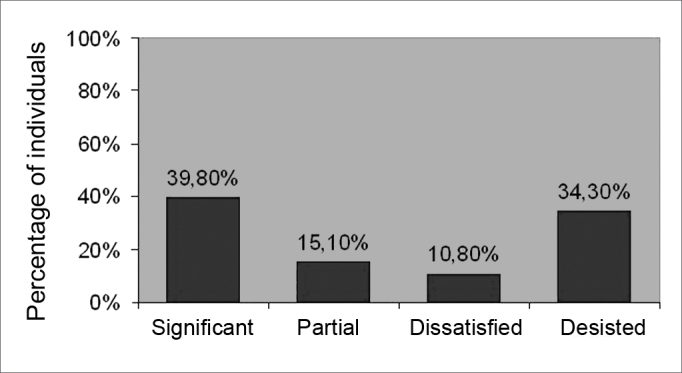
Distribution of subjects according to the result of VR.

Figure 3 shows the results of VR.

Table 5 shows the distribution of subjects that completed the proposed treatment compared to the results of VR (N=61).

**Table 5:** Distribution of subjects that completed the proposed treatment compared to the results of VR (N= 61).

Results of VR	N	%
Significant improvement	37	60,7
Partial improvement	14	22,9
Unsatisfactory	10	16,4
Total	61	100,0

Table 6 shows the distribution of subjects that completed the proposed treatment according to the otoneurological evaluation and the result of VR (N=61).

**Table 6:** Distribution of subjects that completed the proposed treatment according to electronystagmography and the result of VR (N=61).

	Normal		Peripheral		Central		Mixed		Inconclusive		Total	
	N	%	N	%	N	%	N	%	N	%	N	%
Significant improvement	7	11.5 %	21	34.4%	5	8.2%	1	1.6%	3	4.9%	37	60.6%
Partial improvement	–	–	11	18.1%	2	3.3%	–	–	1	1.6%	14	16.4%
Unsatisfactory	3	4.9%	4	6.6%	2	3.3%	–	–	1	1.6%	10	23.0%
Total	10	16.4%	36	59.1%	9	14.8%	1	1.6%	5	8.1%	61	100.0%

Table 7 shows the mean number of session as a function of the otoneurological evaluation.

**Table 7:** Mean number of sessions as a function of electronystagmography.

Electronystagmography	Mean number of sessions
Normal	3.6
Peripheral	4.1
Central	5.9
Mixed	4.0
Inconclusive	4.7

General mean number of sessions: 4.46 (SD= 2.57)

## DISCUSSION

The sex and mean age distribution of the sample (Table 1) are in agreement with data in the literature, which shows a prevalence of dizziness in females.^2^^,^^16^^,^^19^^,^^20^^,^^22^

Audiological results for each ear (Figure 1) revealed that over half of subjects had normal hearing in at least one ear (57.0% in the right ear and 55.9% in the left ear). The most frequent type of hearing loss was the sensorineural type (33.4% in the right ear and 32.4% in the left ear). We would like to mention that we found no classification system encompassing all of the audiometric configurations encountered in the sample. We chose Lloyd and Kaplan's 1978 classification,^15^ which we considered the most appropriate. It was not, however, sufficient for classifying all subjects, as many patients had hearing loss at single or high frequencies.

In the otoneurological evaluation, we found that 60.2% of the sample presented peripheral type electronystagmography, as shown on Table 2.

The analysis of electronystagmography according to sex (Table 2) revealed results that are similar to those presented by Resende et al.,^2^ Simoceli et al.,^16^ Moreira et al.,^19^ Nishimo et al,^20^ and Traldi et al,^22^ who showed the prevalence of altered otoneurological results in females.

An analysis of the time elapsed between the onset of dizziness and the beginning of VR (Table 3) revealed that most of the subjects in our sample (64 subjects −68%) started the proposed treatment 1 to 5 years after the onset of complaints. Our hypothesis is that there are few available vacancies and a significant demand for patients requiring VR at this university hospital. It is a reference hospital that receives patients referred from many other healthcare units. The few available vacancies increase the waiting time.

Figure 2 shows the psychoaffective complaints in the sample. Our results are similar to those found in the literature.^1^^,^^17^^,^^19^ Certain vertigo syndromes may leave patients depressed and insecure. Fear of dizziness may lead to anxiety, and even to panic. Depression may develop and cause individuals to withdraw from social activities, family members and professional colleagues, as well as limit leisure and household activities. It may lead to dependence on family members and may compromise family dynamics.^1^

Our findings on the general health status (Table 4) are similar to those in the literature, in which the causes of vertigo are: benign paroxysmal postural vertigo (BPPV), acute unilateral vestibular disease (vestibular neuritis), Ménière's disease, perilymphatic fistula, bilateral vestibular disease, cardiovascular, metabolic, hormonal and immunological disorders, spinal disorders, head or neck trauma, disorders of vision, generalized neuropathy, decreased cerebral blood flow, and psychological disorders such as panic and anxiety.^4^^,^^5^^,^^6^^,^^20^

About one third of the patients did not comply with the proposed VR treatment, which we considered a high proportion. Thirty-two patients (34.3%) quitted (Figure 3). In more detail, the sample shows that 14 patients (43.7%) went to the outpatient unit only once. Of these, 9 (64.3%) quitted for no apparent reason, 2 (14.3%) considered that they were feeling well and that did not need VR, and 3 (21.4%) quitted because they lived far from the healthcare unit. The remaining 18 patients that quitted the treatment (56.3%) had been to at least two VR sessions; 7 of them (21.9%) reported partial improvement of dizziness in the last session to which they went.

According to our analysis, the reasons for abandoning the proposed treatment were: severe depression, lack of motivation, very poor general health status, and (re)commencement of work, according to the data of Bittar et al.^21^

Of patients that concluded the proposed treatment (61 subjects), 51 patients (83.6%) benefited from VR. Thirty-seven subjects (60.7%) reported significant improvements and 14 (22.9%) reported partial improvements (Table 5). These results confirm VR as an effective treatment for reducing dizziness and improving the quality of life of patients.^1^^,^^3^^,^^20^^,^^21^^,^^22^

The relation between otoneurological evaluation results and VR shows that significant and partial improvement was more evident in patients with peripheral otoneurological alterations (Table 6), which is similar to the findings of Telian and Shepard.^18^ These authors demonstrated that the best results of VR may be seen in patients with incomplete or decompensated unilateral peripheral lesions.

The relation between the mean number of sessions and otoneurological results (Table 7) shows that patients with a diagnosis of central lesions required more sessions (mean −5.9 session). This finding confirms Taguchi's^9^ observation that patients with central lesions respond slowly to therapy and have a poor clinical outcome.

The general mean number of VR sessions was 4.46 (Table 7), similar to the data presented by Pedalin and Bittar,^1^ who reported a mean 4 sessions.

We believe that more systematized data collection and dissemination about patients before, during and following VR will improve the possibility of understanding the successes and failures of this form of therapy, which will optimize the care of these patients.

## CONCLUSION

Patients seen in the Vestibular Rehabilitation Outpatient Unit of the Otoneurology Sector of a university hospital were mostly female (60.2%), with a mean age of 52.82 years (SD=15.0). Over half of the sample had normal audiometry in both ears (57.0% in the RE and 55.9% in the LE). The sensorineural type was the most frequent form of hearing loss. There were more altered otoneurological results in females, of which most were peripheral alterations (in 60.2% of cases). Psychoaffective complaints were frequent.

The mean number of VR sessions was 4.27 (SD= 2.57); patients with central lesions were those that required more sessions (mean 5.9 sessions). The treatment drop out rate reached 34.3%.

Over 83% of patients benefited from VR. Significant and partial improvement was more evident in patients with peripheral otoneurological alterations.

Patients with peripheral otoneurological results had a better prognosis; however, patients with central or mixed conditions also improved with treatment.

We conclude that VR, when well indicated and when patients comply, is an effective therapy within a small number of sessions for the treatment of patients with vestibular disorders.
